# Insect Pest Control from Chemical to Biotechnological Approach: Constrains and Challenges

**DOI:** 10.3390/insects16050528

**Published:** 2025-05-15

**Authors:** Stefano Civolani, Massimo Bariselli, Riccardo Osti, Giovanni Bernacchia

**Affiliations:** 1 Department Chemical, Pharmaceutical and Agricultural Sciences, University of Ferrara, Via Borsari 46, 44121 Ferrara, Italy; 2 Plant Protection Service—Emilia-Romagna Region, Via Andrea da Formigine, 3, 40128 Bologna, Italy; massimo.bariselli@regione.emilia-romagna.it; 3 Department Life Sciences and Biotechnology, University of Ferrara, Via Borsari 46, 44121 Ferrara, Italy; riccardo.osti@unife.it

**Keywords:** plant protection, insecticides, IPM, agrochemicals

## Abstract

The large growth in the global population requires new solutions for the control of harmful insects that compete for our food. Changing regulatory requirements and public perception, together with the continuous evolution of resistance to conventional insecticides, also require, in addition to innovative molecules with different modes of action, new non-chemical control strategies that can help maintain efficient integrated pest management programs. The last 30 years have inaugurated a new era characterised by the discovery of new mechanisms of action and new chemical families. Although European programs also promote a green deal in the crop protection sector, the existing thorough regulations slow down its spread and the adoption of new products. In light of these changes, this review will describe in more detail the dynamics of discovery and registration of new conventional insecticides and the difficulties that the agrochemical industries encounter. Subsequently, the different innovative control strategies alternative to conventional insecticides based on natural substances of different origin, entomopathogenic microorganisms, semiochemical and semiophysical compounds, and classical and augmentative biological control will be described. The advantages of these green strategies will be illustrated and also the constrains to their diffusion and commercialisation. Finally, the main biotechnological discoveries will be described, from transgenic plants to symbiotic control, classical genetic control, and, more recently, control based on insect genomic transformation or on RNAi. These new biotechnologies can revolutionise the sector despite some constrains related to the regulatory restrictions present in different countries.

## Simple Summary

The growing global population intensifies the need for effective insect pest control to safeguard food supplies. Traditional insecticides face challenges due to evolving insect resistance, regulatory hurdles, and shifting public perception, prompting a demand for innovative solutions. Over the past 30 years, new modes of action and chemical families have emerged. In Europe, unfortunately, stringent regulations under the Green Deal framework have slowed their adoption. This review examines the current state of conventional insecticide development, highlighting the difficulties agrochemical industries face in discovery and regulatory approval. It also explores alternative, non-chemical pest control strategies such as natural substances, entomopathogenic microorganisms, semiochemicals, and biological control methods. These environmentally friendly solutions offer advantages for integrated pest management, though their commercial success is limited by market and regulatory barriers. Additionally, the review describes cutting-edge biotechnological innovations, including transgenic crops, RNA interference (RNAi), symbiotic control, classical genetic control, and insect genome editing. While these technologies have the potential to transform pest management, their widespread implementation remains constrained by varying regulatory policies across countries. The future of insect pest control likely lies in a balanced integration of conventional, biological, and biotechnological strategies.

## 1. Introduction

**Figure 1:**
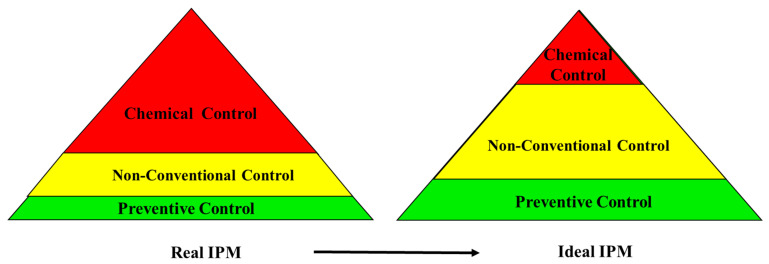
Ideal and real uses of insect pest control strategies in IPM.

The large growth in the global population requires an increase in the quantity and quality of food production. Worldwide, about 18% of crops are lost due to insect damage [1,2], without considering the loss due to plant diseases and weeds. Consequently, insect pest control is an essential tool in agriculture to avoid product losses that would otherwise proceed, from 50% in wheat to more than 80% in cotton production [1,2]. Starting from the 1950s, insect pest control has been based mainly on so-called ‘conventional’ synthetic insecticides, but in the last few decades, an integrated pest management (IPM) strategy has gained a significant role and has allowed, with good intentions and harsh realities, satisfactory results [3,4]. IPM ideally consists of a three-layered pyramidal approach with, at the base, field manipulation and cultural practices and the use of resistant host plants. On the second level, we find biological and microbiological control strategies and the use of semiochemicals and new biotechnological tools. Chemical control is a last resort, with conventional insecticides applied, if necessary (Figure 1). However, the reality is that these three layers are rearranged upside down, with conventional insecticides still remaining the main tools to effectively reduce insect pest damage in many countries (Figure 1). Currently, chemical control and, more generally, the entire crop protection sector suffers from a crisis of innovation—in particular, for herbicides discovery and less for insecticides and fungicides [5], even if, in Europe, most of the new products are not yet registered [5]. In fact, the commercial availability of conventional insecticides has decreased over time, since regulatory requirements in different regions prohibit the use of toxic [6] and environmentally unfriendly chemicals. On the other hand, the registration of new conventional insecticides, even if environmentally friendly, has become more challenging, especially in the EU, due to the same regulations. This should lead to the development of new, non-conventional alternative insect pest controls, but unfortunately, despite the promotion by legislative authorities and new biotechnological discoveries, their approval still encounters difficulties, even in the areas where they are encouraged. The scope of the present review is the description of the different components of IPM, highlighting the interconnections and the innovations and also the limits to their diffusion. We aim to describe how, in the last decades, conventional insecticides, particularly those toxics or banned, have been replaced by new control methods and how effective these methods are, trying to integrate these two approaches in the same analysis.

## 2. Chemical Control

Although conventional insecticides represent the most used solution for the control of insect pests, the worldwide number of active ingredients that can be used is more or less constant [7], even if farmers’ perception is somehow associated with a decline. The causes of this perception are multiple and include the appearance of resistant insects, more stringent regulations, and the subsequent difficulties that agrochemical industries encounter in developing new insecticides [5]. The occurrence of resistant insect pests is strictly linked to the frequent use of insecticides in the field. Since the 1950s, these cases have shown exponential growth (more than 18,000 cases reported), resulting in 362 insecticides that are no longer effective for a specific pest in a specific region [8]. For this reason, a specific technical group called the IRAC (Insecticide Resistance Action Committee) was established in 1984 to provide coordinated responses for the prevention or delay of this phenomenon [9,10].

Concerning regulatory restrictions, these vary between different areas; in particular, registration in the EU is governed by EC Regulation 1107/2009, which replaced the old Directive 91/414 EEC. This regulation guarantees, on the basis of the precautionary principle, a high level of protection for human health and the environment through an in-depth risk assessment before authorisation for sale and use. The regulatory framework on the use of plant protection products has undergone a profound revision with Directive 2009/128/EC, which promotes the sustainable use of plant protection products by reducing their risks and impacts on human health and the environment, promoting the use of integrated pest management and alternative approaches or techniques, such as organic farming and non-chemical plant protection products. This new directive has led to the rapid removal from the European market of many conventional insecticides—unfortunately, in some cases, without adequate replacement with newly registered insecticides or other alternative techniques. The most well-known impact of this directive has been the restriction on many neonicotinoids and fipronil due to their negative impact on bees; a survey conducted by Kathage et al. [11] in eight areas of the EU showed that, in some cases, the banned neonicotinoids have been replaced by pyrethroids, and in other cases, they have not been replaced. This restriction nevertheless was perceived by many farmers as an increase in costs or damages.

**Figure 2:**
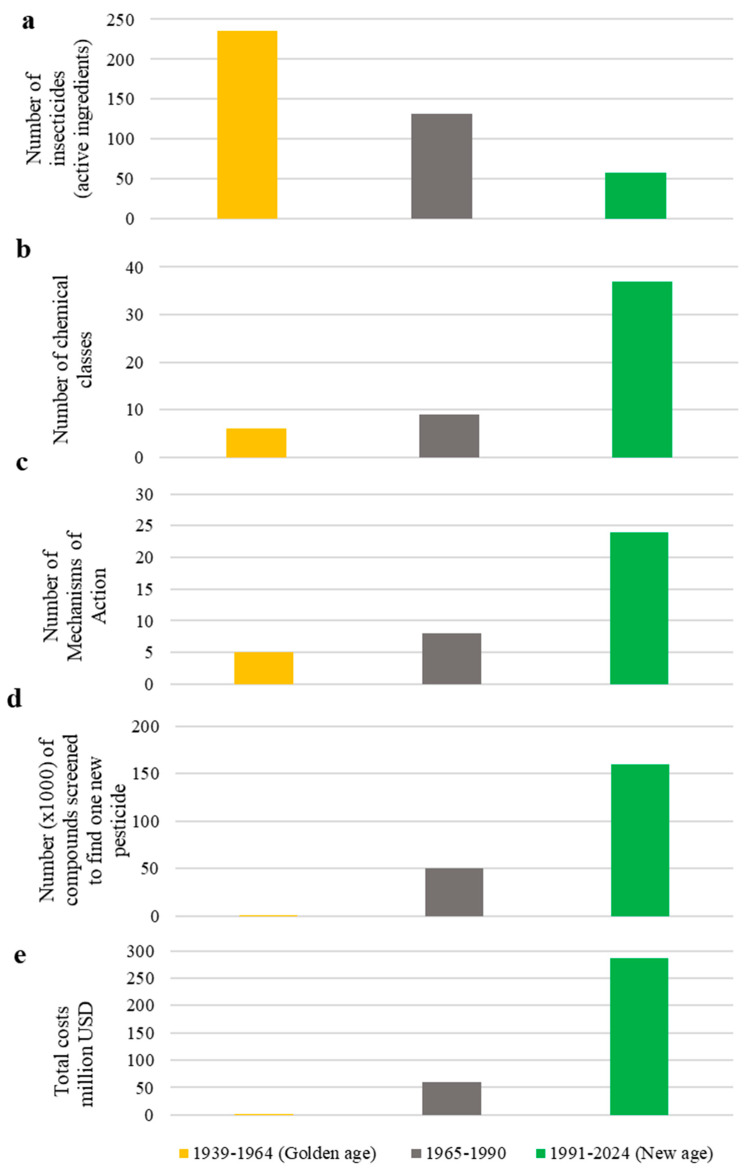
Evolution from the “Golden Age” to the “New Age” in terms of insecticides (**a**), chemical classes (**b**), mechanisms of action (**c**), efficacy on screened compounds (**d**), and costs (**e**) [8,17,20].

The same EU rules have also made more complex and time-consuming the research and development (RD) and registration of new conventional insecticides by the agrochemical industries as compared to previous decades. Historically, research on conventional insecticides began in the 1930s and took off in the late 1940s, mainly by some chemical industries in Switzerland, Germany, and the United Kingdom, which led to the discovery and straightforward registration of products such as DDT, cyclodienes, organophosphates, and carbamates. Their strong effectiveness led to the belief that most of the plant protection problems were considered solved; therefore, this period was called the “Golden Age” [12]. However, after the initial successes, several negative aspects emerged because of the environmental and toxicological issues of the conventional insecticides [13], which some might call the “Dark Age” of insect pest control [14]. These serious issues have forced the agrochemical industries to develop new insecticides, fulfilling the requirement for more environmentally friendly products. As a result, RD and registration costs have risen significantly over time (Figure 2). In 2017, it was estimated that it was necessary to screen 159,000 chemical molecules among the ~10^60^ (total chemical space) [15] to identify one that can aspire to become a marketable pesticide [16]; on the other hand, in the period 1965–1990, this number was down to 50,000 (Figure 2d) [7,17]. Furthermore, the time needed for the RD and registration of an insecticide continues to increase, as, while, at the beginning of the 1990s, it was possible to develop and market a new insecticide in 8 years, now, it takes more than a decade [8]. The economical consequence of these changes is very high: in 1990, 60 million USD was enough to place an insecticide on the market, while, today, these costs fluctuate around 300 million USD [8,17] (Figure 2e). This is one of the reasons why about 70 modest-sized agrochemical companies existing in 1970–1980 in the US, EU, and Japan have subsequently merged into 6 huge multinational companies and 19 small companies in Japan [18,19].

During the Golden Age, the agrochemical industries have registered more than 200 insecticides belonging to a few chemical classes and related to only 5 mechanisms of action (MoAs) or targets (Source: IRAC; Figure 2a–c). On the other hand, from 1991, the so-called “New Age”, there has been the significant identification of 24 new MoAs, targeted by a small number of insecticides (Figure 2a,c) [8,17,20]. Unfortunately, only a few of them have been registered in the world since 2011, mostly outside the European market (Table 1).

**Figure 3:**
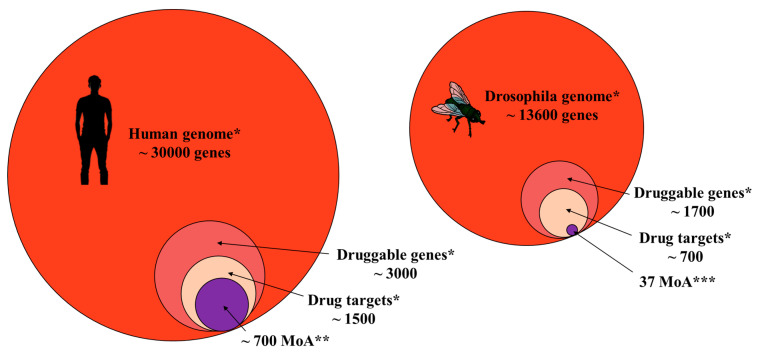
Size comparison between the human and *Drosophila* genomes, druggable genes, drug target, and targets. * [25], ** [27], and *** IRAC 2024.

In a recent review, Swale [24] described the difficulties and delays of the agrochemical industries as compared to the pharmaceutical ones. The number of targets (37 MoAs in total; IRAC 2024) for insecticides cannot be compared to the number of targets for FDA-approved pharmaceutical drugs (700) [24,25,26,27]. Already, thirty years ago, Casida [28] hypothesised that the number of drug targets in insect pests was limited as compared to the counterparts in humans for several reasons, such as the size of the genome, which is larger in humans than in insect pests and therefore richer in targets. In the human genome, it has been calculated that, among the 3000 druggable genes, half of them can be a drug target (Figure 3) [25]. In the model insect per excellence, *Drosophila melanogaster*, among the 1500 druggable genes, only 700 insecticide targets have been predicted [25].

The reason for this limited number of MoAs in insects lies, above all, in the difficulty of identifying new molecules that not only are able to affect the target insect pest, but they also have to fulfil the requirements in terms of environmental safety, low toxicity to humans, low costs, and optimal agropharmacokinetics and dynamics [7,24]. Furthermore historically, the agrochemical industries have often followed “competitor-inspired” and “next-generation” strategies to discover and screen new molecules [5,8,20,29,30]. Unfortunately, these strategies have led to the creation of many conventional insecticides but only a limited number of “first in their class” new products targeting innovative MoAs (25% of the total). On the other hand, strategies such as “bioactive hypothesis” and “natural product-based” have been able to discover many “first in their class” insecticides [5,20,29]. Whitin the “bioactive hypothesis”, the ligand-based approach works through agrochemical-like ideas (chemistry-based, bioactive scaffolds, third-party sources, broad screening, and libraries) and has been more successful in terms of discoveries (28%) than target site-based approaches [29]. It is worth noting that pharmaceutical industries, by focusing on well-known targets and by working on fine adjustments, have been more successful using the latter approach for drug discovery instead of the former [29]. For example, this approach focused on G protein-coupled receptors (GPCRs) has led to the development of 35% of approved human drugs [24]. Among them, there are chemically different small molecules (inorganic and organic) but also biological molecules such as peptides [31]. The agrochemical industry should take up the challenge to achieve the same success [32], even if it is lagging behind the pharmaceutical industry [24]. The only active ingredient used to target insect GPCR receptors, amitraz, belongs to the formamidine chemical family (IRAC MoA 19). A recent work by Gressel [32] highlighted the need to consider not only small molecules [33,34] but also insect peptides to target these receptors, especially because it is currently possible to study in silico the receptor structures and, through new software, predict the putative peptide “ligands” with insecticidal activity [35]. However, for these potential insecticides, there will be many challenges to face in order to reach commercialisation. Natural products are therefore a good source of inspiration for new conventional insecticides not to be used as themselves (which will be described in the next section) but as semisynthetic derivates or synthetic compounds that mimic natural products [29,36]. Overall, natural product derivatives represent only 3% of the total “first in their classes” discovered, while natural product mimics are 18% [29].

## 3. Non-Conventional Control

As mentioned before, in the ideal IPM, a fundamental role is played by non-conventional methods such as natural insecticides, entomopathogenic microorganisms, semiochemicals, and biological and biotechnological controls (Figure 1). These methods are often grouped by different legislations with the term biopesticide or biocontrol agents (BCAs), even if there is no formal agreement on what is a biopesticide. Chandler et al. [37] defined biopesticide basically as an agent obtained from a living microorganism or natural origin able to control plant insect pests and divided in different categories based on the function or source. Different legislations, however, are less clear, and they classify biopesticides in different ways. In the EU, biopesticides or BCAs are divided into natural substances, microorganisms, and semiochemicals, while, in the US, they fall into slightly different types: microorganisms, biochemical products, and plant-incorporated protectants (PIPs) [38]. Despite the growing demand for more sustainable solutions in agriculture (EU Directive 2009/128), in Europe, there is the paradox that the registration of these BCAs differs only minimally from the registration of conventional insecticides. Furthermore, there is no fast track procedure; although these products are characterised by lower toxicity [38], they must still meet the same rigorous safety requirements and efficacy tests as conventional insecticides (EU Regulation 1107/2009). On the contrary, in the US, the entire process lasts approximately 4 years, with a cost in the order of 3–7 million USD [17].

### 3.1. Natural Substances

Natural substances used in insect pest control can be obtained from different sources: plant, animal, microbiological, and mineral.

The natural substances of plant origin are also called “botanicals” and contain secondary metabolites such as phenols, terpenes, alkaloids, and others (Table 2). Historically one of the first botanical used as an insecticide was nicotine, an alkaloid obtained from the leaves of tobacco plants (*Nicotiana tabacum*), known for its properties since 1746 (Table 2). The active ingredient nicotine acts as a modulator of the nicotine acetylcholinergic receptor [39,40] and, unfortunately, is also extremely toxic for mammals, and it is rapidly absorbed through the skin in humans; therefore, interest in its use has been lost over time, with China and India being exceptions. The activity of the extract of *Tanacetum cinerariaefolium* flowers [39,40] has also been known for a long time (1828), due to a set of esters among which pyrethrin I is the most representative active ingredient (Table 2). The action of pyrethrin is to modulate the Na channel. In its pure state, pyrethrin has been considered moderately toxic to mammals, but commercial products containing a limited amount are considerably less toxic. Pyrethrin is particularly labile and breaks easily in the presence of UV. Some studies have shown that the half-life of pyrethrin is approximately 2 h in field conditions, which significantly limits its use against insect pests in agriculture. Nevertheless, pyrethrin is still considered the best-marketed botanical in the world for domestic and structural insect control. Rotenone has been used as an insecticide for over 150 years (1848). It is one of the isoflavonoids produced in the roots or rhizomes of the tropical legumes *Lonchocarpus*, *Derris*, and *Tefrosia* (Table 2). This compound inhibits the mitochondrial complex I electron transport in cellular respiration. A study on rotenone persistence determined that the half-life is 4 days, so the degradation is reasonably fast, but it is more persistent than pyrethrin [39,40]. Due to its toxicity to non-target organisms, the use of rotenone has lost importance in recent years, but it is still used in China and India. A more recent botanical with insecticidal effects is azadirachtin, a limonoid from the neem tree *Azadirachta indica* (Table 2) with well-documented, strong antifeeding, insect growth regulating, and reproductive effects, marketed since 1985 [41]. The complexity of azadirachtin’s molecular structure has precluded its industrial synthesis, and research on simpler mimetics has not led to the development of new insecticides. Applied research has therefore only focused on a variety of natural formulations based on neem seeds easily harvested and extracted [41]. Many people credit azadirachtin, especially its commercial development in the US and Germany, for a renewed interest in botanicals in the 1980s after the advent of conventional insecticides [40]. Azadirachtin’s use is widely approved in many countries, second only to pyrethrin. More recently, interest has shifted to essential oils; in fact, while, about 20 years ago, studies on azadirachtin dominated the scientific literature on botanical insecticides, in the last decade, essential oils have been a trending topic [40]. Essential oils are derived from aromatic plants and are easily extracted by steam distillation of plant materials (Table 2). They contain many terpenes (monoterpenes) and volatile low molecular weight phenols. Essential oils act as insecticides because they induce repellence, growth inhibition, and neurotoxic effects exerted through GABA, octopamine synapses, and acetylcholinesterase inhibition [42]. On the other hand, they are not toxic for mammals (with few exceptions), and they have a short environmental persistence [42]. Thousands of works have been published on the efficacy of essential oils of different origins against different insect pests (3600 publications in the decade 2004–2014, according to [43]), but all this research has led to the registration of a limited number of commercial products [40]. The first essential oil to be registered in 2008 as an insecticide in the US was derived from *Chenopodium ambrosoides*, and it contained a mixture of three terpene derivatives: alpha-terpinene, D-limonene, and para-cymene and has been marketed by Bayer in North America and subsequently in the EU since 2019. The essential oil extracted from orange (*Citrus x sinensis*) peel, mainly containing limonene, was developed in South Africa and has been registered in the US and EU since 2015 [44]. Other essential oils have been marketed as insecticides, such as those derived from rosemary and peppermint and from *Capsicum* oleoresin and garlic (Table 2) and from *Celastrus angulatus* (registered in China). Another type of botanical belongs to the vast group of alkaloids. Their use is rather limited to a few countries. For example, in China, several insecticides have been registered based on matrines and the related quinolizidine alkaloids extracted from *Sophora flavescens* or on veratrines and the related cevadine alkaloids extracted from *Veratrum nigrum*. An insecticide based on these alkaloids (Veratran D^®^) was reintroduced in the US in 2004, while, in India, an insecticide (Anosom^®^) based on the active ingredient squamocin (belonging to the polyketide natural class of acetogenins) extracted from *Annona squamosa* was registered in 2008. Other acetogenins extracted from the *Annona* genus and related genera have been patented for insect control already in the 1980s but, due to regulatory restrictions, have not been marketed either in North America or in Europe [39].

In recent years, plant-derived peptides have emerged as a new promise in the field of plant protection [45]. Among the promising active ingredients, there is certainly pea albumin (b subunit, PA1b), a peptide of 37 amino acids, isolated from the seeds of *Pisum sativum* [46] and *Vicia fabae* [47]. This peptide acts by binding to the V-ATPases of the plasma membrane of the midgut cells of insects [48]. Other peptides of plant origin with insecticidal activity are cyclopeptides or cyclotides containing 28–37 amino acids with three cysteine bonds that modify their shape and increase their stability towards digestive enzymes and high temperatures [49]. For example, in *Clitoria ternatea* (Fabaceae), more than 85 cyclic peptides have been isolated since 2011 [50], and among these, the Cter M (Cliotide T3) of 29 amino acids is the active ingredient of the first plant-based cyclopeptide insecticide (Sero-X^®^) used on cotton and other crops in Australia on important insect pests [51].

Globally, botanicals have had an increased use as a consequence of new authorisations for agricultural use and of domestic consumption [40]; nevertheless, their use has several limiting factors preventing their diffusion or their practical applications. The content of the active ingredients in the raw material shows great variability; furthermore, most botanical insecticides show rapid degradation in the field and, therefore, require frequent applications [52]. Finally, regulatory approval in developed countries is expensive and time-consuming, as it is mainly based on the chemical pesticide approval model [53]. In the EU, it is based on Regulation No. (EU) 1107/2009 and (EU) 528/2012, even if the same substance is listed in the Food Flavourings Regulation (Regulation (EU) No. 1334/2008). Registration is therefore the main obstacle, especially for small agrochemical companies that cannot support the related costs. The EU and its Member States are certainly the most restrictive in this regard compared to other legislations. Currently, there is a large difference in the number of approved active substances, with only 6 in the EU, 11 in India, 13 in the US, and 16 in China. In fact, more than 30 promising botanicals have not been approved under EU PPP regulations since 2007 [54].

Peptides with insecticidal activity of animal origin mainly come from the venom of arthropods (e.g., spiders, scorpions, ants, etc.) and marine animals (e.g., jellyfish, anemones, and snails of the genus *Conus*). Among them, spider venoms are an incredible source of insecticidal peptides, especially those with disulfide bridges (insecticidal spider venom peptide, ISVP) [55,56]. Some of these, such as ω-ACTX-Hv2a (antagonist of insect voltage-gated calcium channels) and the ω-κ-HXTX-Hv1a (Nicotinic Acetylcholine Receptor Allosteric Modulator), are derived from the venom of *Hadronyche versuta* and are putatively active against many insect pests. The latter is the active ingredient of Spear^®^, the first peptide-based insecticide registered in the US in 2018 by Vestaron Crop Protection (Kalamazoo, MI, USA), a recent winner of the Green Chemistry Award.

Insecticides can also be based on natural substances of microbial origin produced by actinomycetes and containing secondary metabolites called polyketides: avermectins and spinosyns. The discovery and development of avermectins in the 1970s earned Ōmura and Campbell the 2015 Nobel Prize in Physiology or Medicine, while the development of Spinosad led to the 1999 Presidential Green Chemistry Challenge Award. For example, Avermectins, produced through fermentation by *Streptomyces avermitilis*, are a group of various macrocyclic lactones; a mixture of avermectin B1 (abamectin) is the active ingredient of Vertimec^®^ or Agrimec^®^, registered for the first time in 1985 in the US [57]. Spinosyns and their analogues are another group of macrocyclic lactones obtained from the fermentation of *Saccharopolyspora spinosa*, and spinosyn A (about 85%) and spinosyn D (about 15%) make up the active ingredient of Spinosad, first marketed (Success^®^, Laser^®^) in 1997 by Dow AgroSciences (Indianapolis, IN, USA) [58].

There are also few promising insecticidal peptides originating from microorganisms such as Destruxin A produced by the fungus *Metarhizium anisopliae*, longibrachin A-I from *Trichoderma longibrachiatum*, and Beauveriolide I and Iso-isariin D produced by the fungus *Beauveria bassiana* [45].

Isman, in his reviews [39,40], highlighted that, while the discovery of insecticides of microbial origin such as abamectin and Spinosad have had considerable commercial success, those of plant origin have had a setback in the last 20 years (Table 2), with the botanicals market lagging significantly behind in comparison to the microbial one [40,59]. This is mainly due to the economical choices made many decades ago by the big agrochemical industries that decided to focus only on some substances instead of others.

### 3.2. Entomopathogenic Microorganisms

Many microorganisms such as viruses, fungi, and bacteria are used directly in plant protection not only against harmful insects but also against plant pathogens, weeds, and, in some cases, as resistance inducers and growth regulators [60]. Research on entomopathogenic microorganisms as alternatives to conventional insecticides is very abundant, and from the work by Hernández-Rosas [61], it emerged that the number of scientific publications since 1973 is continuously growing, with a peak in the late 1990s; however, the registration of these microorganisms is the limiting factor to full practical use, especially where there is more restrictive legislation. The most widely used entomopathogenic bacterium is *Bacillus thuringiensis* (Bt), initially commercialised as a spore-based formulation, called Sporeine^®^, in 1938 in France [62]. At the moment, numerous formulations are available, even if they cover only 2% of the total insecticidal market. *B. thuringiensis*, during sporulation, produces a toxic protein crystal (the Bt δ-endotoxin) classified into two families known as Cry and Cyt. The Cry (from crystal) and Cyt toxins (from cytolytic) are characterised by a general cytolytic activity that causes midgut cells lysis upon ingestion by susceptible insect pests. A third family of protein toxins, the Vip (from Vegetative Insecticidal Proteins), are not classified as crystal toxins, since they are secreted from vegetatively growing cells [63,64,65]. These toxic proteins are host-specific and can cause host death within 48 h. They do not harm vertebrates and are safe to humans, beneficial organisms, and the environment. The sprayable commercial products consist of bacterial spores and δ-endotoxin crystals produced by fermentation, but since 1995, numerous insecticidal Bt-coding genes have been expressed in transgenic crops as plant-incorporated protectants (PIPs) [66]. These Bt transgenic plants are the most important contribution of biotechnology to insect pest control in agriculture, and they are widely used in several countries, covering more than 109 million hectares in 2019, for example [67,68].

While the use of Bt sprays in the field has led to a limited number of cases of high-level resistance in insect pests [69], there are currently 11 insect pest species that have evolved practical resistance to transgenic Bt plants [68,69].

Other microbial insecticides are based on entomopathogenic cytotoxic baculoviruses. In the US and Europe, the *Cydia pomonella* granulovirus (CpGV) is used against *Cydia pomonella* on apples, while, in Brazil, the nucleopolyhedrovirus of *Anticarsia gemmatalis* was extensively used on soybeans in the mid-1990s.

Lastly, the main entomopathogenic fungi used in insect pest control are the ascomycetes *Beauveria bassiana* and *Metarhizium anisopliae*. Brazil is the largest country extensively using commercial biopesticides based on *M. anisopliae* against spittlebugs on sugarcane and grassland.

As already stated, BCAs, and especially entomopathogenic microorganisms, are regulated by EU Regulation 1107/2009, and therefore, their registration requirements differ slightly from chemical substances. The most important differences are that for microbial plant protection products data regarding the origin, the properties, and the survival of the microorganism, and its residual metabolites can be based on the literature if available, especially if the microorganism and its metabolites are not hazardous to humans in the recommended dose [70].

**Figure 4:**
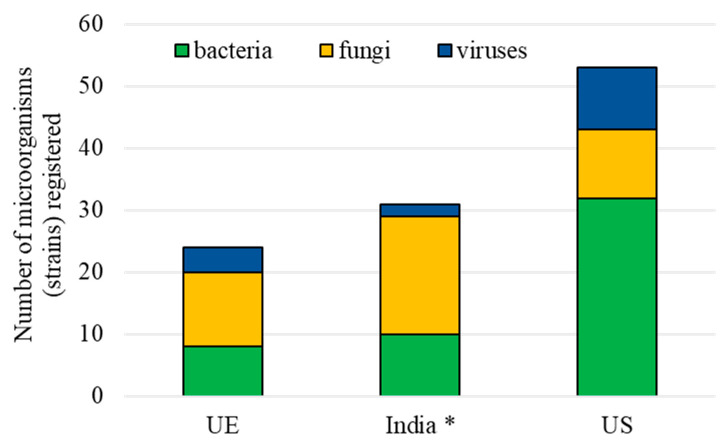
Entomopathogenic microorganisms registered in 2024 (* Data for India are limited to 2017).

In the EU, four regulations have recently been issued (2022/1438, 2022/1439, 2022/1440, and 2022/1441) to simplify the registration procedure and the risk assessments to be conducted by its Member States and the European Food Safety Authority (EFSA) by focusing on the specific biology and ecology of microorganisms. Some aspects are still unclear, for example, the lack of guidelines related to the sensitising power of microorganisms, and in this case, the precautionary principle is applied. Furthermore, it is worth noting that BCAs do not necessarily need efficacy data only in the US registration, while these are requested in other countries [17]. Currently, 52 microbial insecticides are registered in the US [60], while, in 2017, there were 31 microbial insecticides registered in India [71]. In the EU, despite the present difficulties, the overall trend of registered microbial insecticides is currently growing from 12 in 2014 [38,72,73] to 24 insecticides available in 2024 (Figure 4).

### 3.3. Semiochemical

Insects, like all animals, need to communicate to carry out their essential activities, especially reproduction and feeding. Until a few decades ago, it was believed that insects depended mainly or exclusively on smell and taste perceived through chemical signals; therefore, the term semiochemical was coined. Depending on their role, semiochemicals are divided into subcategories: pheromones are used by individuals of the same species, while allelochemicals are used between different species in different ways; kairomones, for example, are beneficial only for the receiver, while allomones benefit only the producer and harm the receiver, and finally, sinomones are beneficial for both the producer and the receiver.

More recently, the role of physical stimuli, such as light, sounds, and vibrations, in insect communication was also discovered, often coupled with the semiochemicals. These signals are called semiophysicals and are divided into functional subcategories [74]; among them, substrate-born vibrations have received much attention, given the high number of published works and some promising applications [75].

Both semiochemicals and semiophysicals can be exploited to interfere with essential insect pest activities to limit their damage to crops [76]. In recent decades, the vast literature on semiochemicals and semiophysicals has resulted only in the registration of a few usable products. Most of the published data concern the application of sex pheromones, often combined with other semiochemicals or semiophysicals, in mating disruption [77], mass trapping [78], and attract and kill [79] and push–pull strategies [80]. The first publication demonstrating the potential of sex pheromones for insect pest control is almost sixty years old [81], and it led to the first product for mating disruption of *Pectinophora gossypiella* registered in the US in 1978. Almost 50 years later, mating disruption is used worldwide. The Internet database “Pherobase” currently lists 149 insect pest species, 133 of which are moths, for which mating disruption techniques have been demonstrated. The first semiochemical product for attract and kill was registered in 2009 in Australia as Magnet^®^ for the control of *Helicoverpa armigera* on corn and cotton. Other products used successfully are 2,4-decadienoate (extracted from pear fruits) (Cidetrak DA Mec^®^, Adair, OK, USA), an attractant kairomone coupled with an insecticide for controlling *Cydia pomonella* in the US, the Spintor Fly^®^ against *Bactrocera oleae*, and Attracap^®^ used for controlling *Agriotes* spp., while Trinet^®^ is used for controlling *Halyomporha halys* [82] and *Popillia japonica* [83] with limited efficacy. Most of the pheromones currently authorised in the EU are grouped under the term “straight-chain lepidopteran pheromones” (SCLPs), corresponding to 18 acetates, 8 alcohols, and 4 aldehydes. Two substances that do not belong to the SCLP group are also approved as pheromones: lavandulyl senecioate used for the control of *Planococcus ficus* in grapevines and rescalure against *Aonidiella aurantii* in citrus fruits. The marketing of SCLPs in the EU requires approval according to Regulation (EC) 1107/2009 and, more recently, Regulations (EU) 540/2022 and (EU) 1251/2022. To facilitate the application and approval of semiochemicals, a guideline (SANTE/12815/2014 rev. 5.2) was published in 2016, subsequently validated in 2018 by the OECD, accompanied by an EPPO guide for the evaluation of efficacy in 2019.

### 3.4. Biological Control

Biological control is based on the use of entomophagous insects (or other macroorganisms) as predators and parasitoids, and it comprises classical biological control and augmentative biological control. Classical biological control is based on the introduction of one or more natural exotic enemies of an invasive insect pest accidentally arrived in an agroecosystem. In the last 130 years, approximately 2000 exotic entomophagous insects have been employed for the control of 165 exotic insect pest species in 196 countries or islands [84,85]. Augmentative biological control, which began in the early 1900s, consists of rearing entomophagous insects in biofactories that are periodically and continually released in the field to counteract the damage caused by the pest. This augmentative biological control has steadily increased in importance since the 1970s because of the spreading of conventional insecticide resistance, and it has been applied both in Europe (using about 170 entomophagous species for the control of about 100 insect pests) [85] and in the world (about 230 entomophagous species in total) [86] but on an inexplicably small surface area (about 0.4% of the cultivated land) [87]. After 2000, a decrease in the number of new entomophagous species occurred, probably because efficient natural enemies were already available for most insect pests and because of more stringent regulations for the import of exotic entomophagous species [88] through the so-called Habitats Directive (Council Directive 92/43/EEC). The same directive prevents the introduction into the EU of all exotic species without exception for potentially useful species, making classical biological control practically impossible. In the following years, some allowances were made, for example, the Italian Government in 2020 allowed the release in the field of the exotic *Trissolcus japonicus* egg-specific parasitoid for the control of *H. halys* [89] and, in 2021, the release of *Ganaspis brasiliensis*, a larval endoparasitoid, for the control of *Drosophila suzukii* [90].

### 3.5. Biotechnological Control

#### 3.5.1. Synthetic Peptides

Natural peptides are among the most-studied molecules for their potential role as insecticides, but this potential is often compromised by the limited bioavailability and biostability that prevents them from reaching the target and their high cost of production. Their linear and bulky nature inhibits their penetration and also makes them easy targets for enzyme degradation. For these reasons, a biotechnological approach has worked on the modification of insect peptides to obtain peptidomimetics—in particular, through a cyclisation process, along with the addition of non-canonical amino acids, making the peptide less bulky and degradable. Alternatively, they can be conjugated or fused to a carrier moiety to facilitate penetration and increase stability.

Many works have been performed on the structural modification of insect neuropeptides. These are usually involved in essential biological processes, such as growth and development, feeding, digestion, excretion, reproduction, metamorphosis, and behaviour [91], and for this reason, they have long been proposed as targets for the control of insect pests [33,92,93]. Since the 1990s, a few authors have suggested the importance of developing cyclised peptidomimetics-based insecticides (backbone cyclic neuropeptide-based antagonist or BBC-NBA) [33,94] but with limited success during the following years. Some promising results were obtained with a kinin analogue [95] and a modified insect short neuropeptide sNPF [96] showing high activity against aphids.

Conjugation can be obtained through an attack on polymers such as PEG, which has been used by the pharmaceutical industry since 1970. This technology has also been studied in insect pest control—in particular, on the decapeptide Trypsin-modulating oostatic Factors (TMOF), which pegylation significantly increases the resistance to enzymatic degradation without preventing their passage through the epithelium, towards the receptor. Their toxicity has been observed on *Aedes aegypti* larvae and on *Heliothis virescens* and *Helicoverpa zea* [97]. Other types of carriers are represented by chitosan and its derivatives, lectins, and small peptides rich in arginine and lysine residues, called Cell-Penetrating Peptides (CPPs), that have the ability to rapidly cross the plasma membrane of gut epithelial cells, acting as vectors for biotoxins bound to them [98]. At the moment, no modified peptide-based insecticide has been registered in contrast to the pharmaceutical field, where, among the 110 or so peptides used, 28 have been cyclised [99] and 25 have been conjugated [100].

#### 3.5.2. RNA Interference

Since the discovery of the RNA interference mechanism in the model nematode *Caenorhabditis elegans* more than two decades ago [101], much work has been done on the use of this technique for the specific control of insect pests [102]. This molecular mechanism, present in almost all organisms, causes the specific gene silencing triggered by a double-stranded RNA (dsRNA), exogenous or endogenous, which is homologous to the target gene. These dsRNAs are cleaved inside the cells into small RNA fragments (siRNAs) of about 21–23 nucleotides. These siRNAs are then incorporated into an RNA-induced silencing complex (RISC) that uses the siRNAs as a template to recognise and degrade the complementary messenger RNA (target gene) [103]. This silencing phenomenon is unfortunately not ubiquitously efficient in the various orders of insects—in particular, it is very effective on Coleoptera, while it is less efficient on Lepidoptera (intermediate and variable values for Hemiptera and Diptera) [104,105,106]. This variability depends on intrinsic factors—in particular, the different cellular uptake and the intestinal degradation [104]—but also on the method of application of the exogenous dsRNA. This homology-based silencing mechanism has the advantage of being highly specific for the target pest, with no interference on non-target organisms. The easier and cheaper way for dsRNA delivery is based on the use of transgenic plants (host-induced gene silencing, HIGS) [107,108] or microorganisms (virus-induced gene silencing, VIGS) [109] as feed for the target pest. Foliar distribution (spray-induced gene silencing, SIGS) [110] can be less effective, especially for sucking or boring insect pests, since the production costs, the uptake, and the stability of dsRNA in the environment need to be optimised [105,111]. Recently, different types of carriers or biomaterials have been tested; liposomes, nanoparticles, nanosheet (Bioclay^®^), chitosan, and other materials can increase the stability of dsRNA in the environment and facilitate the uptake into insect tissues [112]. A further issue is related to the choice in target gene. Usually, silencing is exerted on genes coding for fundamental functions that need to be selected for each insect pest species. In terms of costs of production of dsRNA, in two decades, considerable progress has been made, moving from prohibitive initial costs with chemical synthesis (100,000 USD per gram of dsRNA) to acceptable costs through microbial fermentation and purification processes (1 USD per gram of dsRNA) or cell-free bio-processes (0.5 USD per gram of dsRNA) used by some biotechnology companies (GreenLight, Medford, MA, USA; AgroRNA-Genolution, Seoul, Republic of Korea, and RNAiSSANCE, St. Louis, MO, USA) [109]. After two decades of research and the publication of a very high number of articles, the EPA authorised in 2023 the first RNAi-based insecticide, Ledprona (Calantha^®^), produced by GreenLight, to be applied as a foliar spray (SIGS) containing a dsRNA silencing the essential metabolic gene *LdPSMB5* of the *Leptinotarsa decemlineata* [113]. In the US, these SIGS-based RNAi products are considered “biochemical insecticides” like semiochemicals (Regulation 40 CFR 158.45) and, unlike conventional insecticides, are considered natural compounds. In the EU, no specific category is yet available for these SIGS-based dsRNA products, and their registration still follows the same regulatory framework as classic synthetic insecticides provided for by Regulation (EC) 1107/2009 [114,115,116].

### 3.6. Genetic Control

The first application of genetical control of insect populations occurred 70 years ago with the Sterile Insect Technique (SIT), which then led to the use of Genetically Modified (GM) insects through new biotechnologies. The Sterile Insect Technique, also called autocide, was applied for the first time to *Cochliomyia hominivorax* with the release of radiation-treated sterile males to induce zygote mortality in wild females to progressively reduce the harmful natural population [117,118]. This technique is based on intensive rearing of the target species, sexing, sterilisation, and, finally, on the recurrent release of sterile insects into the field. Sexing is the most critical phase of the entire process, since the release of sterile insects of both sexes is much less effective than releasing only males. A genetic sexing system is preferable, as reported in the case of *Ceratitis capitata* strains (Vienna 7 and 8) which females carry two recessive phenotypic markers, white pupae (*wp*), and temperature-sensitive lethal (*tsl*) [119], that facilitate their fast removal from the rearing population. Other improvements have been obtained over time with genetic transformation; in this case, it was possible to overcome some critical issues, such as the reduction of fitness of the irradiated insect and the separation of the sexes. The first genetic transformation of an insect was achieved in 1982 in *D. melanogaster* using the P element transposon as the vector, which was followed 10 years later by the transformation of *C. capitata* using Minos transposon. In the following years, the first practical applications of the insect pests *C. capitata* (2012, Morocco), *Bactrocera oleae* [120], and *Plutella xylostella* (2017, US) (under study in *D. suzukii*) were conducted by the company Oxitech (Abingdon, UK) using the conditional dominant lethal expression system or RIDL^®^ (Release Insects Dominant Lethal). By introducing a female-specific dominant gene, this system allows easy sexing under rearing conditions and the subsequent offspring lethality in the field conditions after the release of the transgenic males [121]. In the last two decades, with the advent of genome editing—in particular, with the discovery of CRISPR/Cas9 in 2012—it has been possible to also obtain site-specific disruption (INDELs caused by non-homologous end joining, NHEJ) or gene insertion (homology-directed repair, HDR) in insects. In a recent report, Yan et al. [122] described several CRISPR applications, such as the creation of engineered sexing strains in *C. capitata* through the introduction of *wp* and *tsl* genes on the sex *transformer* gene, improving efficiency and duration over time compared to classical genetic sexing. It is also possible to improve classical SIT through CRISPR precision guided insertion of the genes linked to female sex determination and male fertility. With this CRISPR-based strategy, for example, developed on *D. suzukii*, the low fitness of irradiated males created in classical SIT is avoided, and excellent results have been obtained in field cages [123]. Another strategy is the sex ratio distortion technique (CRISPR–sex ratio distortion) that disintegrates the X chromosome with the targeted insertion of an *I-PpoI* gene in spermatozoa and consequently allows only male offspring. A breakthrough was recently described with the “gene drive” technique (CRISPR-based gene drive) that promotes super-Mendelian inheritance of a self-propagating desired gene (selfish “gene drive”) that spreads through a population faster than traditional Mendelian inheritance by means of gene drive-carrying males. This application can be used to target genes that control female fertility or vitality, leading to the collapse of the entire target population within a few generations after the release of these transformed males. The first work on CRISPR/Cas9-mediated gene drive in insects was published in 2015 on *D. melanogaster*. Subsequent experiments have been successfully performed not only on different mosquito species but also on insect pests such as *D. suzukii*, *C. capitata*, and *P. xylostella* [124], but these are still far from the field [125]. Although these new genome editing technologies have several advantages over conventional control methods, some issues arise related to ethical problems and public perception [126], regulatory obstacles [127], and complicated ecological interactions that must be carefully evaluated before starting the release of these genetically modified insects [128].

The success of all these genetic control techniques often depends on extensive territorial planning, an “areawide” management (AW-IPM) [129]. Pérez-Staples et al. [130] recently reported that, worldwide, particularly in some subtropical countries of Central and South America, many AW-IPM programs have been successful in controlling several insect pests, particularly tephritid dipterans flies such as *C. capitata* and *Anastrepha ludens*. Even for Lepidoptera, despite their genetic resistance to radiation [131], successful SIT programs have been reported. However, the same authors cited cases of malfunction and abandonment due to several reasons, both technical and field-related, or interruptions due to a lack of funds, as happened for *Anastrepha obliqua* in Mexico [130]. Recent promising research on invasive species such as *D. suzukii* [132] and *H. halys* [133] should also be mentioned.

In addition to the Sterile Insect Technique (SIT), there is also a variant called the Infected Insect Technique (IIT) [134]. Inside the insect body, there are microorganisms called reproductive manipulator symbionts that facilitate the growth of symbiont-infected females to the detriment of the males or the uninfected females. Among these microorganisms, the best known are *Wolbachia pipientis*, *Candidatus* Cardinium hertigii, *Rickettsia* spp., *Spiroplasma*, and *Arsenophonus nasoniae*, and the first is undoubtedly the most studied. *Wolbachia* is a genus of intracellular and obligate bacteria, mainly found in the reproductive tissues, observed for the first time in 1924 in the ovaries and testes of the *Culex pipiens* mosquito. *Wolbachia* manipulates the reproduction of the hosts by shifting the sex ratio of the infected species in favour of the female to ensure their own survival. The evolved mechanisms are different depending on the *Wolbachia* strain and the reproductive strategy of the host, and among these, we have the induction of parthenogenesis, feminisation, male killing, and cytoplasmic incompatibility. The latter can be exploited to induce insect pest sterility in field populations using an areawide approach like SIT. This technique involves the release of males infected by *W. pipientis* that mate with uninfected wild females to generate non-viable offspring. The first successful IIT application was achieved in Myanmar, where the target population of the filariasis vector *Culex pipiens* was almost eradicated, and it was also used against insect pests such as *C. capitata* [135], *Rhagoletis cerasi* [136], and *D. suzukii* [137].

An assessment of the existing regulatory frameworks for SIT and IIT has recently been done by Kapranas et al. [138], even if there are no clear guidelines. Regulation and approval for transgenic insects obtained by CRISPR fall within the GM regulations in Brazil, Canada, and the US, while, in the EU, the United Kingdom, and in other countries, these GM insects are prohibited (https://crispr-gene-editing-regs-tracker.geneticliteracyproject.org/#jet-tabs-control-1401, accessed on 30 April 2025).

### 3.7. Symbiotic Control and Paratransgenic Insect

Symbiotic associations between insects and microorganisms have a substantial impact on the host, influencing its nutrition, physiology, ecology, and reproduction. In particular, insect pests often feed on very unbalanced diets and live in very different environments, and for this reason, they establish positive symbiosis with some microorganisms able to provide essential components. Understanding the role of these symbiotic microorganisms can help to develop innovative insect pest control methods [139] through three strategies. The first strategy aims to prevent the vertical transmission (between mother and offspring) of primary symbionts, in particular the extracellular ones mainly present in the gut. The best-known case studies are those concerning *H. halys* and *B. oleae* [140]. The symbiont of *H. halys* is “*Candidatus* Pantoea carbekii”, which, located inside the crypts of the midgut, can provide amino acids, vitamins, and cofactors to the host. Transmission to the offspring occurs through the smearing of maternal secretions released on or near the egg masses during oviposition; the newly hatched nymphs are immediately able to acquire these bacteria. Transmission can be interrupted by sterilising the egg masses, which will later suffer high mortality [141,142] with different products (unfortunately not commercially available). For example, in Italy, Dentamet^®^ was temporarily authorised for foliar spray on *H. halys* egg masses. The symbiont of *B. oleae* is “*Candidatus* Erwinia dacicola”, an obligate symbiotic bacterium strictly necessary for nitrogen metabolism for both adults and larvae that feeds on olives. The symbiotic bacterium has been identified in various organs of the insect pest, and it also affects fertility and offspring survival and is transmitted to the eggs via the ovipositor. A reduction in transmission could have a great impact on the insect pest population. There are also examples with fungicidal or copper-based products able to reduce the transmission of the symbiont, but no insecticidal product is registered as such [140].

The second strategy involves the introduction in the target insect pest of symbiotic strains from a different species to create a heterologous association. For example, in *D. suzukii*, secondary symbionts such as *Tatumella punctata* can be replaced by competition with bacteria from *D. melanogaster*, causing a reduction in egg deposition and larvae fitness [143]. The microorganism replacement can also reduce the permanence of some plant pathogens within the insect vector. For example, recent studies conducted with the model vector *Euscelidius variegatus* (instead of *Scaphoideus titanus*) have shown a lower acquisition of the Flavescence dorée phytoplasma by individuals colonised by the Asaia SF15.14 strain, isolated from a female *Anopheles stephensi,* rather than the native one. This strain is, in fact, able to produce a polysaccharidic biofilm in the vector midgut and to activate the immune response through the Raf gene, therefore limiting the spread of the phytoplasma [144]. Although many studies on these symbiotic control strategies are currently under way, no commercial products are currently available.

The last strategy is the use of paratransgenic insects in which their symbionts have been genetically modified to express molecules that interfere with the transmission of plant pathogens. This symbiotic control was used for the first time in 1991 to combat the Chagas disease in Latin America caused by *Trypanosoma cruzii* and transmitted to humans by *Rhodnius prolixus*. The first experience in plant protection was conducted on *Homalodisca vitripennis*, a vector of *Xylella fastidiosa*, on grapevines. *Pantoea agglomerans*, an endophytic bacterium of grapevines and symbiont of *H. vitripennis*, was modified to express antimicrobial peptides (AMPs; melittin and scorpine-like molecule, with cytolytic and apoptotic activity) active against *X. fastidiosa*, making *H. vitripennis* unable to transmit the plant pathogen [145]. For the AMPs to be effectively released from the GM *P. agglomerans*, it is necessary to introduce the hemolysin excretion system from *Escherichia coli*. In the future, new metagenomic data on the interaction between symbionts, insect pests, and insect vectors might facilitate the development of these control methods within the IPM.

### 3.8. Insect Pest-Resistant Transgenic Plants

The use of insect pest-resistant plants is the basis of an ideal integrated defence and, together with cultivation techniques, is part of preventive control (Figure 1), but unfortunately, classical plant breeding has not been very successful in the development of insect pest-resistant cultivars. How to maintain high productivity and quality, along with insect pest resistance, has subsequently become the dilemma of classical genetic improvement. The transfer of insect pest resistance traits to improved varieties is unfortunately slow and therefore very expensive, because very often, their inheritance is polygenic, while plant disease resistance traits are usually monogenic. Furthermore, the few insect pest resistance traits with monogenic inheritance give a protection that does not last very long due to the appearance of resistant variants in the target pest. Classical breeding for insect pest resistance historically began with the release of the first wheat cultivar (cv. Underhill) resistant to *Mayetiola destructor* in 1780 and continued during the last century to improve resistance on several crops such as maize, rice, and wheat, despite limited resources, for example, the maize breeding lines with polygenic resistance to *Spodoptera frugiperda* developed in the 1970s and now used to combat the recent invasion of *S. frugiperda* in sub-Saharan Africa. A pivotal contribution to classical genetic improvement has been provided by the advent of molecular markers-assisted selection (MAS) in particular to obtain rice varieties resistant to the various biotypes of *Nilaparvata lugens* [146,147].

A radical change came from the innovative discovery by Van Montagu, Schell, and Dell Chilton of the *Agrobacterium tumefaciens* ability to transfer genes to plants in 1974 who received the World Food Prize in 1983. This technique led to the first commercial insect pest-resistant transgenic maize, cotton, potato, and tobacco plants, in 1995 in the US, expressing the *cry* toxin of *B. thuringiensis*. Although 271 Cry proteins are currently known, each active against a specific range of insect pests, counter-resistant insect pest populations have appeared, in particular, several cases of resistant *Helicoverpa zea* were found in maize and cotton from 2002 to 2020 [148]. To mitigate the spread of these phenomena, specific anti-resistance techniques have been adopted, such as High Dose Refuge (HDR) and the pyramiding of various toxins [66]. Other toxins from *B. thuringiensis* such as Cyt and Vip [149] or from other microorganisms [150,151] are being studied to be combined with Cry. More recently, the pyramiding of the Cry3Bb1 toxin with the dsRNA sequence against the *Snf7* gene (a vacuolar trafficking protein) was obtained by Bayer (Monheim am Rhein, Germany) in the SmartStax PRO^®^ maize transgenic line against *Diabrotica virgifera* [152]. Unfortunately, no *B. thuringiensis* toxins are available against Hemipteran insect pests; therefore, research has tried to modify Bt toxins to change their specificity towards these insect pests [146,153]. On the other hand, rice and tobacco transgenic plants expressing lectins or proteinase inhibitors showed low efficacy against Hemiptera and did not reach the market [153,154]. Douglas, in his review [146], outlined new promising control strategies based on transgenic plants, for example, the fusion of insecticidal toxins to a carrier domain to enhance the targeted delivery. The spider toxin Hv1a was fused to the lectin from *Galanthus nivalis* (agglutinin, GNA) or the luteovirus capsid (Pea Enation Mosaic Virus, PEMV) carrier proteins and showed higher activity and conferred resistance to Lepidoptera and Hemiptera insect pests [146,155]. In a further example, the neuropeptide allatostatin of *Manduca sexta* was fused to the GNA, causing mortality in *Lacanobia oleracea* larvae [156].

Furthermore, additional control strategies could be based on GM plants overproducing secondary metabolites, for example, *Arabidopsis thaliana* plants expressing a terpene synthase (S-linalool/(3S)-Enerolidol synthase, *FaNES1*), isolated from *Fragaria×ananassa*, accumulated 40 to 60 times more linalool than the control and showed a total repellence of *Myzus persicae* [146]. Although this and other similar studies have provided excellent laboratory results, they did not reach the practical application in the agricultural field, because high levels of secondary metabolites have negative effects on plant growth and on the final consumer.

An alternative strategy to engineer plant insect pest resistance is to specifically modify plant genes linked to susceptibility to insect pests, the so-called *S* genes, by CRISPR editing. An example is rice, where the cytochrome P450 gene *CYP71A* that catalyses the conversion of tryptamine to serotonin has been mutated to suppress serotonin production and to increase salicylic acid and insect pest resistance [157]. In another study, the overproduction of anthocyanins in tobacco changed the colour of leaves from green to red, deterring *Spodoptera litura* and *Helicoverpa armigera* [158]. Similarly, the modification can be applied to decrease kairomones volatiles that attract their specific pest or to increase synomone volatiles that attract their natural enemies [159].

The current European regulatory framework for the growth of genetically modified organisms is based on Directive 2001/18/EC, implementing the Cartagena Protocol. The directive defines, on the basis of the precautionary principle, through an evaluation of potential adverse effects on human health and the environment, the common procedure for all Member States for the authorisation to release a GM into the environment [160].

Furthermore, in 2018, the European Court of Justice (ECJ) suggested that organisms altered by means of site-directed mutagenesis like CRISPR/Cas9 should also be included in the definition of a GM organism [161].

According to the Directive 2015/412/EU, a Member State has the right to decide whether or not to authorise the cultivation of GM crops in its territory. During the authorisation procedure of an application, a Member State may request that the geographical extension be changed in such a way that its territory is not affected by such authorisation.

The US does not have a specific law regulating GM organisms, but they are assessed under the health, safety, and environmental laws that also apply to conventional products. Therefore, the approval of biotechnology products currently falls primarily under the jurisdiction of three regulatory agencies: the Food and Drug Administration (FDA), the US Department of Agriculture (USDA), and the Environmental Protection Agency (EPA) within the Coordinated Framework for Regulation of Biotechnology [160,162]. In other countries, it is quite common that the regulation and approval follow a case-by-case process [162].

### 3.9. Transgenic Entomopathogenic Microorganisms

Despite the availability of entomopathogenic microorganisms for IPM for several years, their large-scale application is still hampered by several notable factors—in particular, their short term persistence compared to a traditional insecticide and their narrow host range. With the advent of biotechnology, genetic manipulation has become the best solution to improve these aspects [163]. In particular, entomopathogenic bacteria [164], Baculovirus [165], and entomopathogenic fungi [166] have been modified to produce and release recombinant biotoxins or dsRNAs (VIGS) to improve the control of different insect pests through long-term persistence and efficacy. However, social concerns about the release of such GM microorganisms into the environment have led to various controversies, along with doubts, on the effects on non-target organisms, gene flow to wild species, and the occurrence of resistance in target pests. Currently, none of these GM microorganisms are marketed and used in the field, despite the vast bibliography.

## 4. Conclusions

Non-conventional strategies for insect pest control have, in recent decades, led to strong innovations in crop protection to compensate for the withdrawal of old and dangerous conventional insecticides. The continuous discovery of new eco-friendly insecticides, especially with new mechanisms of action, has met with difficulties, especially in the EU, due to the lack of registration because of stringent regulations. In the EU, moreover, the same regulations to promote innovative strategies and non-conventional products limit their registration and diffusion. Furthermore, much research has been carried out on natural biopesticides at an academic level but with limited use in the field, and in the past, this was also due to a lack of interest by the big, multinational agrochemical industries. Recent applications in the biotechnological field, such as RNAi and DNA editing, could give a further boost to innovation in this sector, providing new specific and eco-friendly control methods to be combined into the IPM. For all these strategies to be effectively integrated into pest control programs, a careful balance must be struck between scientific innovation, ecological responsibility, and regulatory clarity.

## Figures and Tables

**Table 1:** New insecticides registered from 2011 [21,22,23].

Active Ingredient	Chemical Class (Nr of Insecticides per Class)	MoA (IRAC)	Commercial Name	Company	Date and Country of 1st Registration	EU Registration
spirotetramat	Tetronic and Tetramic acid derivatives (5)	23	Movento^®^	1	2011 Japan	Yes
cyantraniliprole	Diamides (5)	28	Benevia^®^ Exirel^®^	2	2012 Argentina, 2013 Canada	Yes
sulfoxaflor	Sulfoximines (1)	4C	Closer^®^	2	2013 USA	Yes
flupyradifurone	Butenolides (1)	4D	Sivanto^®^	1	2014 Guatemala and Honduras, (2015 USA and Japan)	Yes
pyflubumide	Carboxanilides (1)	25B	Danikong^®^	3	2015 Japan	No
cyclaniliprole	Diamides (5)	28	Teppan^®^	4	2017 Japan	No
pyrifluquinazon	Pyridine azomethine derivatives	9B	Colt^®^	3	2017 Japan	No
triflumezopyrim	Mesoionic compounds (3)	4E	Pexalon^®^	2	2017 India	No
afidopyropen	Pyropenes (1)	9D	Inscalis^®^	4, 5	2018 Australia, India	No
flometoquin	Phenoxy-quinoline (1)	34	Finesave^®^	6	2018 Japan	No
flupyrimin	Pyridylidenes (1)	4F	Lydia^®^, Emylia^®^ Kevuka^®^	7	2019 Japan	No
fluxametamid	Isoxazoline	30	Gracia^®^	8	2019 Japan	No
acynonapyr	Acynonapyr	33	Danyote^®^	9	2020 Japan	No
benzpyrimoxan	Benzyloxypyrimidines	Unkown MoA	Orchestra^®^	3	2020 Japan, (2021 India)	No
broflanilide	Meta-diamides (3)	30	Exponus^®^	7, 4	2020 Australia, (2021 USA)	No
spiropidion	Tetronic and Tetramic acid derivatives (5)	23	Elestal^®^	10	2020 Guatemala	No
tetraniliprole	Diamides (5)	28	Vayego^®^	1	2021 USA	No
dimpropyridaz	Pyrazole carboxamide (1)	36	Efficon^®^ Cimegra^®^	4	2022 Australia	probable
oxazosulfyl	Ethyl sulfones (1)	37	Alles^®^	11	2022 Japan	No
isocycloseram	Meta-diamides (3)	30	Plinazolin^®^	10	2023 Brasil	No
fenmezoditiaz	Mesoionic compounds (3)	4E	Prexio^®^	4	2025 India	No
spidoxamat	Tetronic and Tetramic acid derivatives (5)	23	Plenexos^®^	1	probably 2025	Not known

1: Bayer, Monheim am Rhein, Germany; 2: Corteva, Indianapolis, IN, USA; 3: Nihon Nohyaku, Tokyo, Japan; 4: Ishihara Sangyo Kaisha, Osaka, Japan; 5: Meiji Seika Pharma, Tokyo, Japan: 6: Nippon Kayaku, Tokyo, Japan; 7: Mitsui Chemical, Tokyo, Japan; 8: Nissan Chemical Industries, Tokyo, Japan; 9: Nippon Soda, Tokyo, Japan; 10: Syngenta, Basel, Switzerland; 11; Sumitomo, Tokyo, Japan.

**Table 2:** Natural substances of plant origin.

Extract Origin	Main Active Ingredient	Product Name	Company	Date and Country of 1st Registration	UE Registration
*Nicotiana tabacum*	nicotine			1940	no
*Lonchocarpus*, *Derris*, *Tefrosia* spp.	rotenone			1947	no
*Tanacetum cinerariifolium*	pyrethrin I			1950	yes
*Azadirachta indica*	azadiractin	Margosan O ^®^	1	1985 USA	yes
*Veratrum sabadilla*	veratrine and related cevadine alkaloids	Veratran D^®^	2	(1961) 2004 USA	
*Annona squamosa*	acetogenine	Anosom^®^	3	2008 India	no
*Chenopodium ambrosoides*	terpinene, limonene, cymene	Requiem^®^	4, 5	2008 USA	yes
*Celastrus angulatus*	celanguline and related dihydroagarofuran sesquiterpenes	Celangulin ^®^	6	2010 China	no
*Capsicum* and Garlic	capsaicin and allicin	Captiva^®^	7	2014 USA	no
*Citrus x sinensis*	limonene	Prev-AM^®^,	8	2015 South Africa, USA	yes
Rosmary and peppermint	geraniol	Ecotec^®^	9	2016 USA	no
*Clitoria ternatea*	Cter M (Cliotide T3)	Sero-X^®^	10	2017 Australia	no
*Sophora flavescens*	matrine and related quinolizidine alkaloids	CE Matrine SL^®^	11	2018 China	no
*Veratrum nigrum*	veratrine and related cevadine alkaloids	CE Veratrum Rhizome Extract ^®^	11	2021 China	no

1: Vikwood Industries, Sheboygan, WI, USA; 2: MGK, Minneapolis, MN, USA; 3: Agri Life, Hyderabad, India; 4: Codena AgraQuest, Davis, CA, USA; 5: Bayer, Monheim am Rhein, Germany; 6: Xinxiang Dongfeng Chemical, Shenzhen, China; 7: Gowan, Yuma, AZ, USA; 8: Oroagri International, Groningen, The Netherlands; 9: Brandt, Springfield, IL, USA; 10: Innovate Ag., Wee Waa NSW, Australia; 11: Chengdu Newsun, Chengdu, China.

## Data Availability

The original contributions presented in this study are included in the article. Further inquiries can be directed to the corresponding authors.
